# In silico ADMET profiling of Docetaxel and development of camel milk derived liposomes nanocarriers for sustained release of Docetaxel in triple negative breast cancer

**DOI:** 10.1038/s41598-023-50878-8

**Published:** 2024-01-09

**Authors:** Kousain Kousar, Shaheer Shafiq, Syeda Tahira Sherazi, Fareeha Iqbal, Usman Shareef, Salik Kakar, Tahir Ahmad

**Affiliations:** 1 Industrial Biotechnology, Atta-Ur-Rahman School of Applied Biosciences, National University of Sciences and Technology, Islamabad, Pakistan; 2 Department of Gynecology, Unit 4, Sir Ganga Ram Hospital, Lahore, Pakistan; 3 Shifa College of Pharmaceutical Sciences, Shifa Tameer E Millat University, Islamabad, Pakistan; 4 Pak-Austria Fachhochschule: Institute of Applied Sciences and Technology, Khanpur Road, Mang Haripur, Khyber Pakhtunkhwa Pakistan

**Keywords:** Biological techniques, Biotechnology, Cancer, Computational biology and bioinformatics, Oncology

## Abstract

This study aimed at encapsulation of commonly administered, highly cytotoxic anticancer drug Docetaxel (DTX) in camel milk fat globule-derived liposomes for delivery in triple negative breast cancer cells. Prior to liposomal encapsulation of drug, in silico analysis of Docetaxel was done to predict off target binding associated toxicities in different organs. For this purpose, the ADMET Predictor (TM) Cloud version 10.4.0.5, 64-bit, was utilized to simulate Docetaxel’s pharmacokinetic and physicochemical parameters. Freshly milked camel milk was bought from local market, from two breeds Brella and Marecha, in suburbs of Islamabad. After extraction of MFGM-derived liposomes from camel milk, docetaxel was loaded into liposomes by thin film hydration method. The physiochemical properties of liposomes were analyzed by SEM, FTIR and Zeta analysis. The results from SEM showed that empty liposomes (Lp-CM-ChT80) had spherical morphology while DTX loaded liposomes (Lp-CM-ChT80-DTX) exhibited rectangular shape, FTIR revealed the presence of characteristic functional groups which confirmed the successful encapsulation of DTX. Zeta analysis showed that Lp-CM-ChT80-DTX had size of 836.6 nm with PDI of 0.088 and zeta potential of − 18.7 mV. The encapsulation efficiency of Lp-CM-ChT80 turned out to be 25% while in vitro release assay showed slow release of DTX from liposomes as compared to pure DTX using dialysis membrane. The in vitro anticancer activity was analyzed by cell morphology analysis and MTT cytotoxicity assay using different concentrations 80 µg/ml, 120 µg/ml and 180 µg/ml of Lp-CM-ChT80-DTX on MDA-MB-231 cells. The results showed cytotoxic effects increased in time and dose dependent manner, marked by rounding, shrinkage and aggregation of cells. MTT cytotoxicity assay showed that empty liposomes Lp-CM-ChT80 did not have cytotoxic effect while Lp-CM-ChT80-DTX showed highest cytotoxic potential of 60.2% at 180 µg/ml. Stability analysis showed that liposomes were stable till 24 h in solution form at 4 °C.

## Introduction

Breast cancer is a type of cancer which is very diverse in terms of disease pathology, clinical behavior, histologic type, and response to anticancer treatment. For last 15 years, breast cancer has been classified based on the expression of certain hormone receptors, namely progesterone receptor (PR), estrogen receptor (ER) and anti–human epidermal growth factor receptor 2 (HER2). Almost one and a half decades back, a new term was coined, namely triple negative breast cancer (TNBC) owing to lack of all hormone receptors ER, HER2 and PR on cancer cells^1^. TNBC accounts for 15–20% of all breast cancers in women. It is therapeutically challenging because of its highly aggressive nature and low response to conventional therapeutic treatments. This cancer is more common in women under 40 years of age (4.2%). TNBC responds well to neoadjuvant chemotherapy, but once it enters metastatic stage, it is very lethal despite different therapeutic modalities being targeted against it^2^.

Docetaxel, which is a taxane derivate is a promising chemotherapeutic drug and has been used in treatment of non-small lung cancer, ovarian cancer, triple negative breast cancer and ovarian cancer. Docetaxel (DTX) is classified as class IV drug that shows poor permeability (P-glycoprotein substrate) and aqueous solubility (6–7 µg/mL in water), these aspects lead to low oral absorption and ultimately low bioavailability of drug. Also, DTX is a substrate for drug efflux pump P-glycoprotein (P-gp), which is an ATP-dependent pump responsible for transporting compounds from the intracellular environment to extracellular space. Activation of P-gp suppress accumulation of chemotherapeutic drugs inside cells ultimately leading to rapid clearance and drug resistance^3^. A drug delivery system would help enhance uptake and accumulation of drug, and will help in achieving desired therapeutic effects of Docetaxel at low doses. Common solubility agents such as Cremophor EL and Tween80 are used to enhance solubility of poorly water-soluble anticancer agents^4^.

Liposomes have proved to be an excellent drug delivery system as they are biocompatible and biodegradable, mimic biological membranes, may incorporate both hydrophilic and lipophilic drugs and can protect the loaded cargo from external degradation. Drugs loaded in liposome carriers can circumvent drug efflux p-glycoprotein transporters and therefore can achieve required concentration as liposome mediated drug deposition interacts well with P-gp efflux pump^5^. Also, liposome carriers above 100 nm are less likely to be cleared by reticuloendothelial system (RES) which consist of cells originating from monocytes, and therefore can remain longer in systemic circulation^6^.

Camel milk has been exploited in human health due to its super beneficial nutritional values and therapeutic effects. Phospholipids constitute the major constituent of milk fat globular membrane (MFGM) derived from Camel milk. MFGM consists of three layers, that includes glycolipids, polar lipids and proteins. The structure consists of an inner triacylglycerol (TAG) core, surrounded by glycerophospholipids and membrane proteins, which are derived from the endoplasmic reticulum, and external bilayers derived from the apical membrane.The MFGM liposomes have a higher degree of saturation of fatty acids which confers them increased stability as compared to liposomes from other sources^7^.

**Figure 1:**
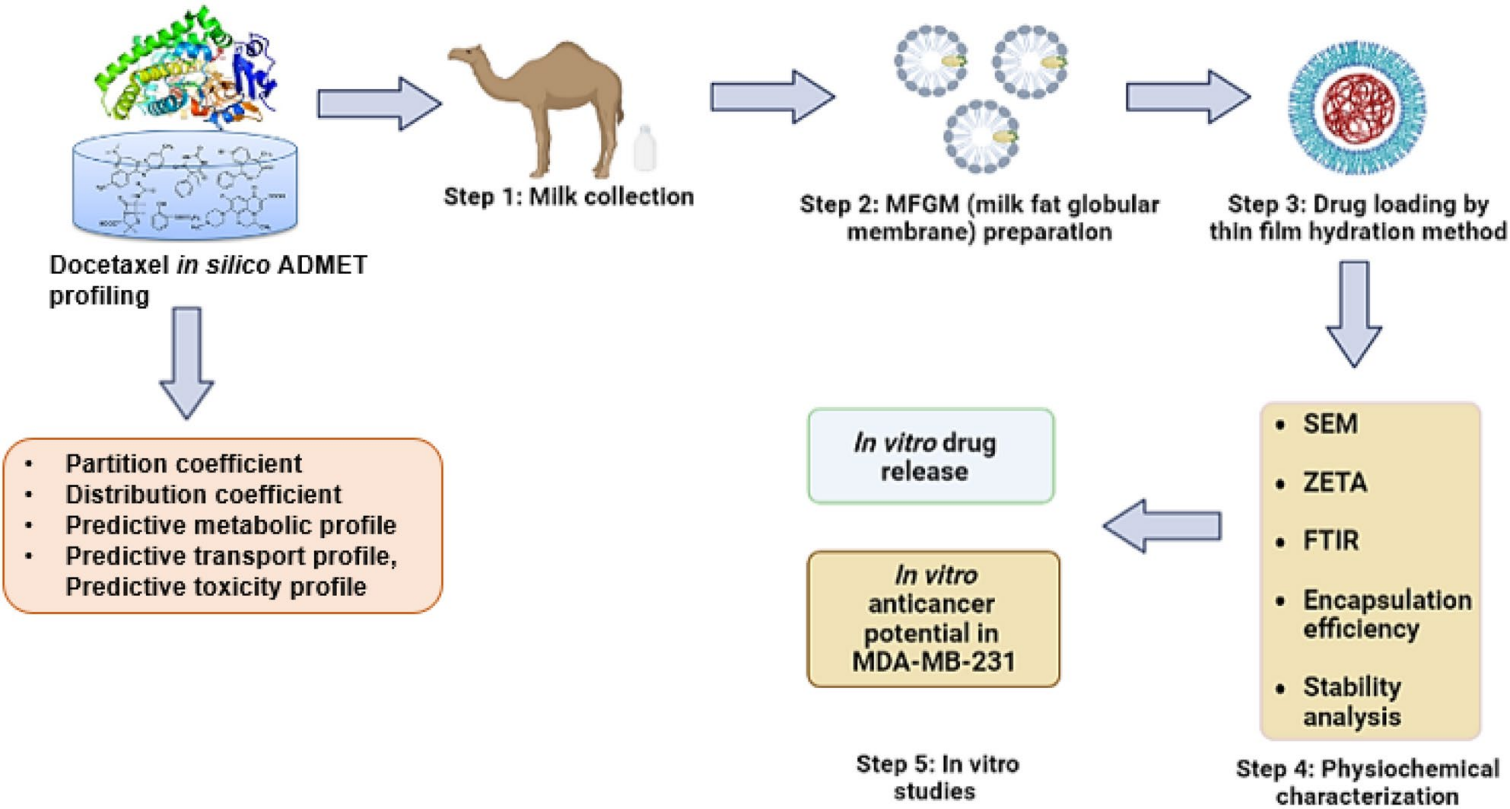
Schematic illustration of research for DOX AMDET profiling and encapsulation of DOX in camel milk derived MFGM liposomes for treatment against TNBC.

The present study aims at encapsulating lipophilic drug DTX in phospholipids extracted from camel milk MFGM derived liposomes, for delivery of DTX to triple negative MDA-MD-231 breast cancer cells. These DTX loaded lipid carriers (Lp-CM-ChT80-DTX) were prepared by thin film hydration method, followed by characterization prior to in vitro anticancer activity. While, in silico analysis was also performed to predict toxicities of DOX against various organs owing to its off-target binding. Figure 1 represents the schematic illustration of this study.

## Materials and methods

### Material collection

Freshly milked Camel milk was bought from the local market. Other chemicals of analytical grade used in this experiment including ethanol, methanol, chloroform, used in this research purchased from Sigma Aldrich USA. MDA-MB-231 cells were kindly provided by Dr. Saeed Khan, Dow University of Health Sciences, Karachi. Docetaxel drug was purchased from Shifa Hospital Pharmacy,Islamabad.

### Docetaxel ADMET prediction

Predictive ADMET studies is the latest exploratory area in drug discovery. The aim is to constitute computational models that relate structural alterations with changes in response, utilizing databases of ADMET data associated with structures. Using these predicted models, therapeutic entities with superior characteristics can be predicted and produced. These databases also enable researchers to extrapolate animal in vivo and human in vitro to predict human ADMET features^8^. These databases give useful information regarding already existing therapeutic molecules as a way to develop them into effective dosage forms. For this study the cloud version of ADMET Predictor (TM) version 10.4.0.5, 64-bit edition was used to predict several parameters about Docetaxel (Pubchem ID: 148124).

### Synthesis of MFGM derived Docetaxel loaded liposomes

#### a) Milk Collection and storage

Camel milk was bought from suburbs of Islamabad where two species Brella and Marecha are common. Camel milk (1000 mL) was collected from 5–10 camels. These milk samples were collected in sterilized 1L bottles and immediately transferred to a temperature-controlled ice box. In lab, 0.1% sodium azide was added to avoid any contamination of samples and microbial growth. All samples were stored in 50 ml Falcon tubes until further use^9^.

#### b) MFGM extraction

During MFGM extraction, the temperature of milk samples was maintained at 2–10 °C. To separate the milk cream, milk samples were centrifuged at 4200 rpm for 30 min, the separated creamy layer was then transferred to a 50 mL falcon tube. To quantify milk fat, 1 M solution of PBS was added, then kept at 37 °C water bath for 30 min with constant shaking to melt milk fat. These samples were centrifuged twice with at 4200 rpm for 30 min with PBS to remove residual proteins. After washing, milk fat samples were transferred to a falcon tube and stored for overnight at 2–10 °C^7^. The next day, samples were homogenized on a vortex mixer at 50 Hz (Hz) for 4 min after mixing with sterile plastic beads. Samples were again warmed using water bath at 37 °C for 15 min and centrifuged at 4200 rpm for 30 min to separate MFGM from yellow fat layer. Fat layer was discarded and white pellets that remained in falcon tube consisted of MFGM, which was weighed and stored at − 20 °C until required^10^.

#### c) Encapsulation of docetaxel in MFGM

Thin film hydration method was used for preparation of docetaxel-loaded liposomes. For this purpose, MFGM was dispersed in chloroform. Docetaxel was dissolved in anhydrous ethanol and mixed with MFGM-chloroform solution in a 250 mL round bottom flask. The solution was evaporated using rotary evaporator at 35 °C till the time a thin film was obtained^11^. This thin film was washed with PBS followed by hydration for 3 h. Following hydration, the MFGM dispersion was sonicated using bath sonicator and docetaxel-loaded liposomes (Lp-CM-ChT80-DTX) were obtained. The prepared liposomes were lyophilized and stored at 4 °C until further use^12^. Same method was used for preparation of empty liposomes (Lp-CM-ChT80) skipping the drug loading step.

### Physiochemical characterization

To analyze size, morphology, functional group characteristics and successful encapsulation of DOX in liposomes, following analysis were performed.

#### a) Evaluation of zeta potential and size distribution

For zeta analysis, the liposomes were dispersed in PBS. The average nanoparticle size and size distribution of Lp-CM-ChT80 and Lp-CM-ChT80-DTX was obtained by using dynamic light scattering zeta nano ZS (Malvern instrument.Co.Ltd). The analysis of diluted formulation (1:10) was done with a scattering angle of 90° at 25 °C. Five different batches were run to obtain an average value and for calculation of standard deviation for average size, zeta potential and PDI^13^.

#### b) Morphological characterization

Scanning electron microscope (SEM) analysis was done using MIRA3 (TESCAN,Czech Republic) to evaluate the morphological characteristics, surface chemistry and microstructure of Lp-CM-ChT80 and Lp-CM-ChT80-DTX. A small amount of lyophilized sample was fixed on SEM stub (aluminium) followed by gold coating. Prior to analysis, excess powder was removed by taping gently^14^.

#### c) FTIR

In order to determine the successful encapsulation of drug and functional groups on surface of MFGM lipid carriers. FTIR analysis was performed using FTIR spectrometer (Nicolet 740SX FTIR spectrophotometer, Madison, WI, USA). Sample in lyophilized powder form was used to determine characteristic peaks of functional groups on Lp-CM-ChT80 and Lp-CM-ChT80-DTX and to confirm successful encapsulation of DTX inside liposomes^15^.

#### d) Drug entrapment efficiency

The amount of DTX loaded into MFGM liposomes was calculated by using spectrophotometer. Measuring concentration of drug in supernatant is an indirect way to calculate the amount of drug loaded in NPs and to calculate encapsulation efficiency of nanocarrier^16^. For this reason, MFGM-DTX solution was centrifuged at 10000 rpm for 1 h, and then supernatant was collected. Using syringe filter, supernatant was filtered and then tested using UV visible spectrophotometer (NanoDrop 2000c, Thermo Scientific, Wilmington, DE, United States) at 290 nm. The following formula was used for calculation of drug loading and entrapment efficiency^17^.

$$\mathrm{Encapsulation\,Efficiency }\left(\mathrm{\%}\right)=\frac{\mathrm{Total\, Drug}-\mathrm{Untrapped\, Drug }}{\mathrm{Total\, Drug}}\mathrm{x }100\mathrm{\%}$$

#### e) In vitro Drug release assay

To analyze the in vitro release of DTX from liposomes, drug release assay was performed by using dialysis membrane. Dialysis membrane bag was immersed in PBS at pH 7.4 and 37 °C. 5 mg of MFGM-DOX lyophilized powder was dissolved in PBS and added to dialysis membrane bag. 1.5 mL of sample was collected after specific time intervals of 0, 4, 8, 24, 48, 72 h and analyzed at 296 nm using UV–Visible spectrophotometer (NanoDrop 2000c; Thermo Scientific, Wilmington, DE, United States)^8^.

### In vitro anticancer activity

In vitro anticancer activity was analyzed by cell morphology analysis and MTT assay using triple negative breast cancer cell line MDA-MB-231. For this purpose, cells were cultured in DMEM media with 10% FBS (Gibco) and 1% pen/strep (100 units of penicillin, 100 µg of streptomycin Gibco). Cells were incubated in a humidified atmosphere of 5% CO_2_, 95% air at 37 °C. Cells were grown in 75cm^2^ tissue culture flasks and later used for analysis after they reached log phase of growth (Jalilian et al., 2021).

#### a) In vitro experimental groups

Three groups were formed for in vitro experiment on MDA-MB-231 cells:

- Group 1: Control MDA-MB-231 cells in DMEM medium,
- Group 2: Lp-CM-ChT80 treated MDA-MB-231
- Group 3: Lp-CM-ChT80-DTX treated MDA-MB-231 cells

#### b) Cell morphology analysis

Detachment of an adherent cell line and changes in morphology of cell like blobbing, aggregation, rounding, and shrinkage are all hallmarks of apoptosis. MDA-MB-231 cells were analyzed for changes in morphology after treatment with concentrations of 80 µg/ml, 120 µg/ml and 180 µg/ml of Lp-CM-ChT80 and Lp-CM-ChT80-DTX for a period of 24 h at 37 °C, 5% CO_2_ and 95% air^17^.

#### c) MTT cytotoxicity assay

MTT is a colorimetric assay based on the potential of living cells to convert water soluble [3-(4,5 dimethylthiazol-2-yl)-2,5-diphenyltetrazolium bromide into insoluble Formazan crystals by activity of dehydrogenases inside mitochondria of live cells. MDA-MB-231 cells were plated in a 96 well plate at seeding density of 1 × 10^6^ (100 μl) cells per well. The cells were incubated for 24 h at 37 °C with 95% air and 5% CO_2._ Cell media was refreshed after 24 h followed by addition of 100 μl of Lp-CM-ChT80 and Lp-CM-ChT80-DTX at concentrations of 80 µg/ml, 120 µg/ml and 180 µg/ml, while control cells were treated with 0.1% DMSO only. On next day, 10 μl of MTT dye solution was added and cells were left to incubate for 4 h at 37 °C and 5% CO_2_. Following incubation, MTT dye was aspirated and 100 μl of DMSO was added to dissolve MTT crystals, cells were again incubated for 1 h. After 1 h, the colored solution in wells was analyzed using microplate reader at 560 nm using (BioTek, Winooski, VT, United States). The following formula was used to calculate cellular toxicity^17^.

$${\text{Cell}}\;{\text{viability}}(\% ) \, = \frac{{{\text{OD}}\;{\text{of}}\;{\text{treated}}\;{\text{cells}}{-}{\text{OD}}\;{\text{blank }} \times {1}00}}{{{\text{OD}}\;{\text{of}}\,{\text{control}}\;{\text{cells}}{-}{\text{OD}}\;{\text{of}}\;{\text{blank}}}}$$

#### d) Trypan blue exclusion assay

The membranes of living cells are intact while dead cells have damaged and perforated membranes. Trypan blue exclusion assay was conducted to analyze the quantity of living and dead cells in the cell suspension. For this purpose, MDA-MB-231 cells were seeded in a 96 well plate at density of 13 × 10^4^ per well. The cells were exposed to Lp-CM-ChT80 and Lp-CM-ChT80-DTX at concentrations of 80 µg/ml, 120 µg/ml and 180 µg/ml and left for 24 h. After 24 h of treatment cells were collected using Trypsin–EDTA (Gibco-New York) for 5 min. Cell palette was collected by centrifugation at 800 rpm for 4 min. 50 µl of cell suspension was mixed with 50 µl of 0.4% trypan blue (Gibco, USA) followed by incubation for 5 min. The mixture was transferred to hemocytometer for manual counting of living and dead cells in suspension. Living cells appeared as white and dead cells appeared as blue as the damaged membrane of dead cells take up the dye. The following formula was used to calculate percentage of living cells in cell suspension:

$$\% \;{\text{Viability}}\;{\text{of}}\;{\text{cells }} = \frac{{{\text{Viable}}\;{\text{cell}}\;{\text{count}} \times {1}00}}{{{\text{Total}}\;{\text{number}}\;{\text{of}}\;{\text{cells}}}}$$

### Stability analysis

Stability of empty and DTX loaded liposomes was analyzed by observation of changes in physical appearance of liposomes suspension. Both Lp-CM-ChT80 and Lp-CM-ChT80-DTX were stored at temperature of 25 °C and 4 °C till 48 h followed by stability analysis.

### Ethics approval and consent to participate

All methods were carried out in accordance with relevant guidelines and regulations.

## Results

### Predictive physicochemical values

Understanding physiochemical properties of a molecule is indispensable to develop an effective dosage form. The initial dose for docetaxel used in this study was 1 mg/kg and the predictive values were measured at both 7.4 pH value. The negative LogP and Vd show that the drug is highly hydrophobic in nature. The predictive BBB (blood brain barrier) value shows that the drug is not able to penetrate through the blood brain barrier very well which may be due to its very large molecular weight 807.88 g/mol.

#### a) Log p (partition coefficient)

**Figure 2:**
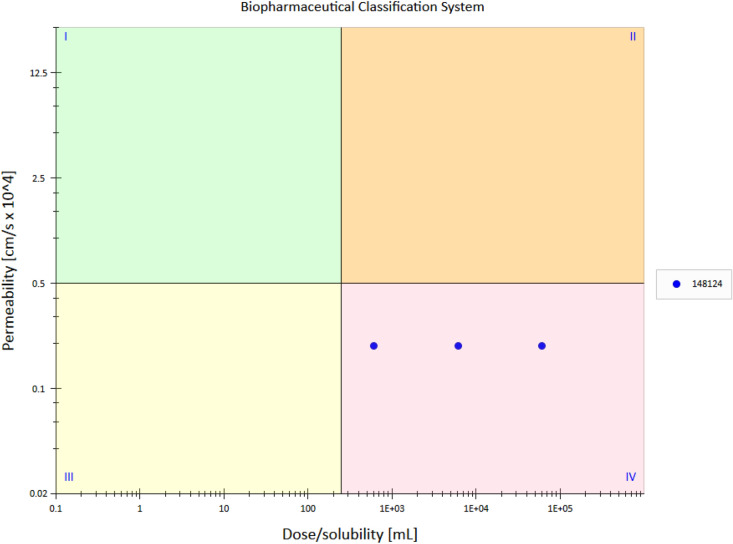
The BCS classification of docetaxel. The three blue dots represent the mg/mL concentration of docetaxel which is 25 mg/ml, 50 mg/ml and 100 mg/ml. Docetaxel showed poor profile on all three concentrations.

According to the solubility and LogP value docetaxel belongs to BCS class IV Drug as shown in Fig. 2. This class of drug exhibits unfavorable characteristics such as low permeability, low solubility, high presystematic metabolism and efflux transport etc. These factors make oral delivery of these drugs very problematic.

The model also showed that the water solubility of docetaxel is very low (0.009 mg/mL) which may cause the drug to not effectively reach the target site. Because blood is the main transporter in body and blood is indeed aqueous in nature. However, in the present study we have converted docetaxel into a liposomal preparation which has significantly overcome this issue. The predictive physicochemical profile of Docetaxel is shown in Table 1.

**Table 1:** Predictive physiochemical profile of anticancer drug Docetaxel.

Parameters	Predictive value	Explanantion
MlogP	0.459	It indicates the Moriguchi's estimation of log P which yielded an RMSE/MAE of 0.93/0.70
S + logP	2.902	This Log P simulation plus model represents the cumulative LogP for the given compound
S + Acidic_pKa	11.25	The value is an indication that predominant acidic groups appear to have an impact on the macroscopic assessment of pKa values
Solution factor	1818.483	Universal salt solubility factor based on S + Sw model
Vd	3.369	The value is a representative of volume of distribution of any drug at steady state concentration
S + Sw	0.009	The simulation plus model predicts the water solubility by the use chemical properties of the substance and is expressed in mg/mL
Diffusion coefficient	0.425	In nonelectrolytes, the water diffusion coefficient at infinite dilution (cm^2^/s × 10^5^) is predicted using the Hayduk-Laudie model
S + MDCK-LE permeability assay	Low	Permeability is classified as low or high using the MDCK permeability classification model, which was developed using ECCS data from Varma et al
S + logD	2.902	According to the model the value represents the predicted log D, at 7.4 pH,
BBB_Filter	Low (97%)	Based on S + logP, the log D at pH 7.4 calculates the probability of passing through the blood–brain barrier with an accuracy of 92%
Permeation skin	72.656	Predicts the human skin permeability profile with units cm/s × 10^7^
S + CL_Metab	No	It demonstrates if metabolism plays a major role in the drug's principal clearance mechanism
S + CL_Renal	Yes	It indicates if the renal pathway is involved in the drug's primary clearance mechanism
S + Peff	0.197	Effective human jejunal permeability (cm/s × 10^4). RMSE/MAE = 0.31/0.25 log units

#### b) Log D (distribution coefficient) and pKa macro constants

**Figure 3:**
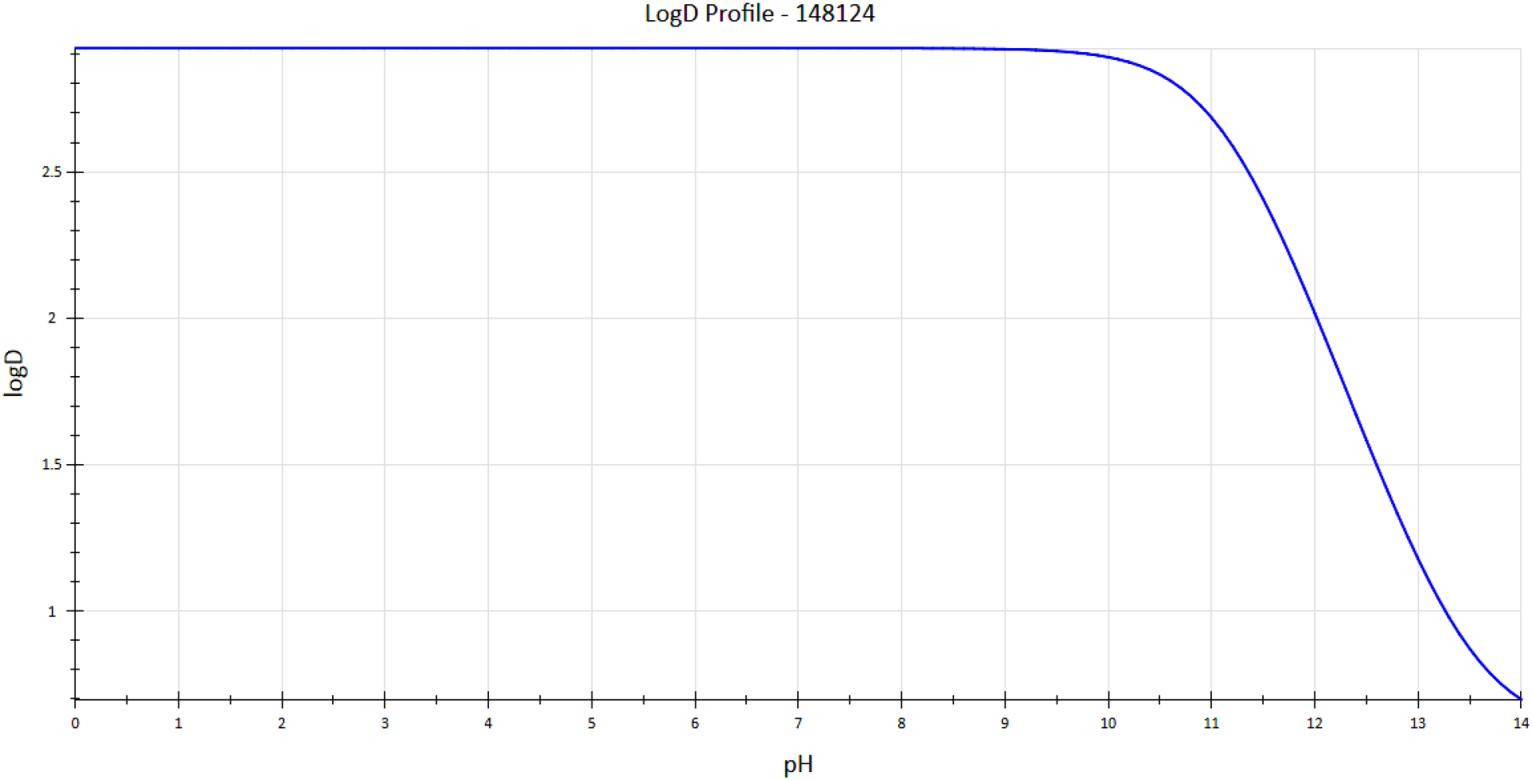
The change in LogD value with rise in pH.

According to the predicted logD values for DOX, the logD rises as pH rises and reaches its maximum between pH ranges of 5 and 14 (Fig. 3). This implies that we can successfully improve the distribution of DOX in the human body by adjusting the pH. But it's crucial to take into account the drug's pKa values, which indicate how much of the substance is accessible at a given pKa value. At physiological pH, higher lipophilicity is correlated with lower pKa values^18^. Figure 3 shows the relationship between LogD and pH.

**Figure 4:**
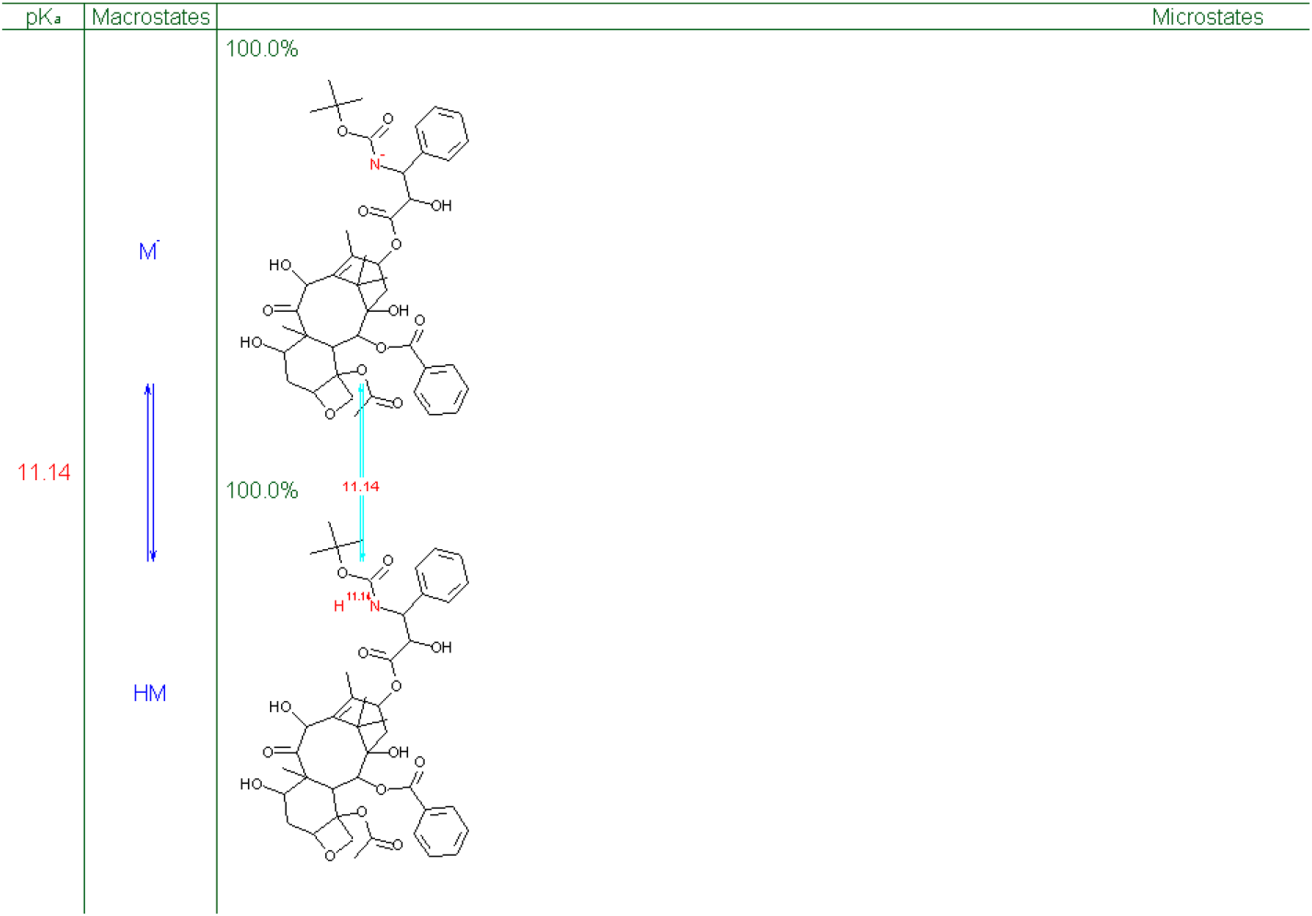
The macrostates, pKa and percentage of drug available at varying pKa values. The red coloured values are for the macroconstants.

Figure 4 shows that the drug is 100% accessible at the target location at a pKa value of 11.14. However, the pH of cancer cells is normally 6.8, which is somewhat acidic and presents a problem. There is a paradox between pH and pKa because lower pKa values maximize medication availability at the target site while raising pH improves Log D. To combat this, we used a liposomal formulation of the drug in our study, which guaranteed the drug's highest concentration and best possible effectiveness at the cancer site. This strategy is in line with the requirement for a successful cancer treatment plan to balance drug lipophilicity, pH, and pKa concerns.

#### d) Predictive metabolic profile

**Figure 5:**
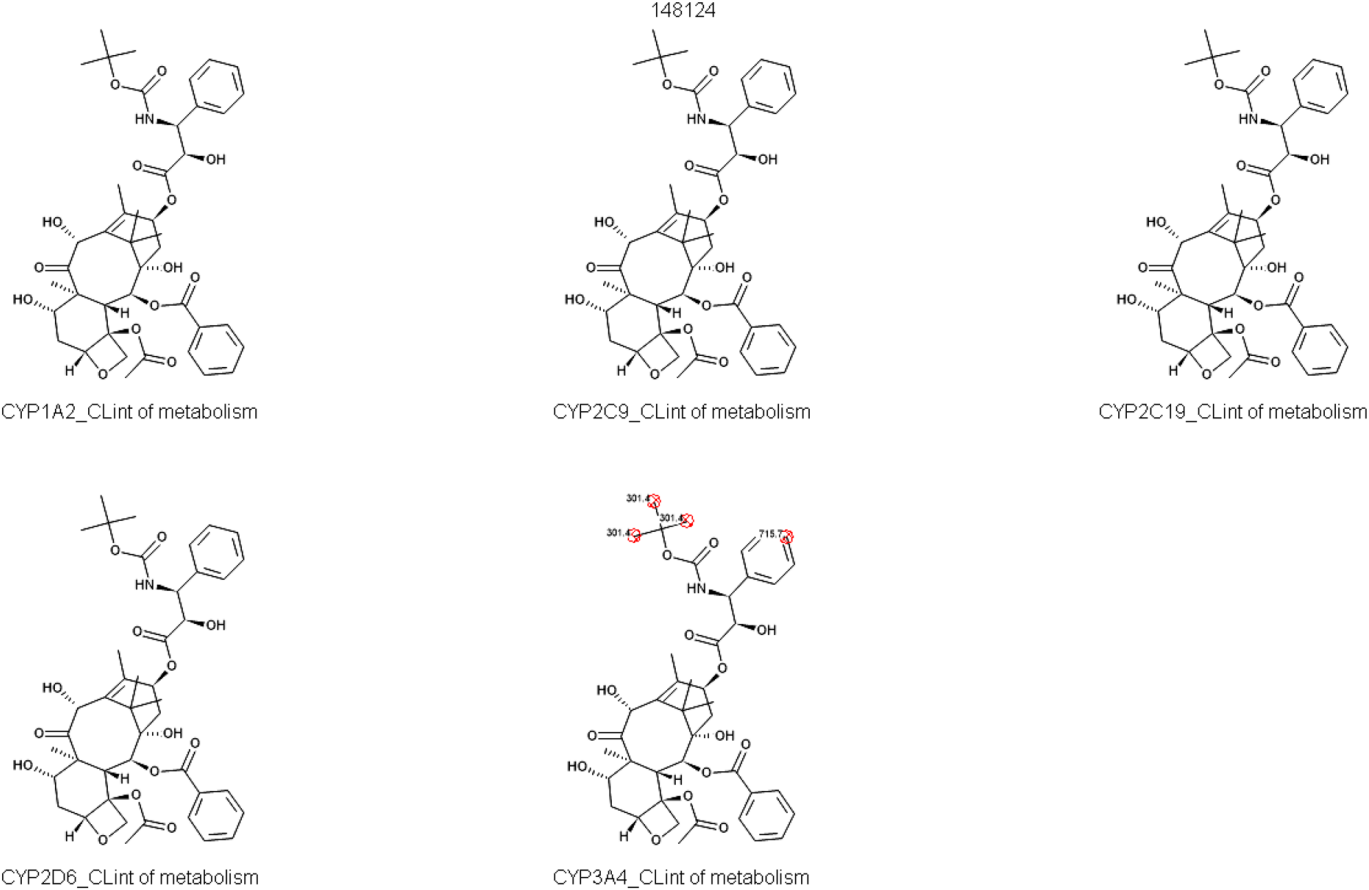
The metabolic susceptibility of docetaxel to a set of enzymes belonging to cytochrome p450 family. The figure shows that docetaxel is only prone to metabolism by CYP3A4. The sites of metabolism are shown in red circles.

The study of drug metabolism is important for a variety of reasons. These include identifying novel compounds by identifying active metabolites, mitigating safety risks associated with the production of hazardous or reactive metabolites, guaranteeing that human metabolites are adequately represented in animal models, assisting in the estimation of human dosages, and comparing the preclinical metabolism of animals and humans^19^. Figure 5 shows susceptibility of Docetaxel to interact with cytochrome p450 enzyme family.

Table 2 shows how docetaxel interacts with several enzymes. Based on the existing evidence, a hypothesis has been put forth that DOX inhibits CYP2C19 and CYP3A4, which may have an effect on the metabolism of other drugs taken concurrently. Furthermore, CYP2C19-induced inhibitory response showed a V_max_ value of 25.000 M/min. However, the study found that the main enzymes that metabolize DOX are CYP2C19 and liver microsomes, indicating that DOX serves as a substrate for these particular enzymes.

**Table 2:** Interaction of DOX with different metabolic enzymes.

Parameter	Predicted value	Explanation
CYP1A2 inhibition	No (70%)	With a 94% prediction accuracy overall, it determines whether the molecule has the potential to be a human CYP 1A2 inhibitor. With regard to the inhibitory potential of the drug molecule, this evaluation establishes the probability that it will impede the CYP1A2 enzyme
CYP1A2 Clint	Non-substrate	For the given substrate, the model yields the expected CYP1A2-mediated oxidation (uL/min/mg HLM protein), which represents the aggregate intrinsic clearance of the protein at the atom level
CYP3A4_Clint	1572.259	Illustrates the expected CYP3A4-mediated oxidation (uL/min/mg HLM protein), which indicates the combined intrinsic clearance at the atom level for that particular protein
CYP3A4_HLM_Km	1.895	The oxidation reaction in unbound form within human liver microsomes, specifically mediated at the atomic level by CYP3A4, is the subject of the Michaelis–Menten constant, or Km. The substrate concentration in enzymatic processes at which the reaction rate reaches half of its maximal value is represented by this measurement, which is expressed in micromoles per litre (µM) Micromoles per litre (µM) is the unit of measurement for the Michaelis–Menten constant, or Km
CYP3A4_HLM_Vmax	0.052	The Michaelis–Menten constant, or Vmax value, is a measurement made in nanomoles per minute per milligramme of HLM protein and is particularly associated with the oxidation reaction that takes place in human hepatic microsomes. The activity of CYP3A4 facilitates this enzymatic process A unit of measurement for the Michaelis–Menten constant is nanomoles per minute per milligramme of HLM protein, or Vmax
CYP3A4_Inh	Yes (80%)	Predicts whether or not the compound is human CYP 3A4 inhibitor (Yes/No). Overall accuracy = 78%
CYP3A4_Inh_testo	Yes (84%)	Qualitative estimation of specific inhibitory properties (Yes/No) against CYP 3A4 mediated metabolism of testosterone. Overall accuracy = 100%
CYP3A4_Sites	C36(938); C37(938); C38(938); C28(647)	Sites of CYP 3A4 mediated oxidation (FIRST step only!). Specific sites shown only if the appropriate substrate model predicts 'Yes' (this behavior can be changed). Numbers in parentheses are propensities of metabolic oxidation for comparing atoms in THE SAME molecule and for THE SAME enzyme only!
CYP3A4_Substr	Yes (98%)	Predicts whether or not the compound is human CYP3A4 substrate (Yes/No). Overall accuracy = 82%
CYP3A4_Vmax	76.565	The Michaelis–Menten constant, or Vmax value, is a measurement made in nanomoles per minute per milligramme of protein and is particularly associated with the oxidation reaction that takes place in human hepatic microsomes. The activity of CYP3A4 facilitates this enzymatic process A unit of measurement for the Michaelis–Menten constant is nanomoles per minute per milligramme of HLM protein, or Vmax
HEP_hCLint	28.548	Predcited human hepatic clearance value in µL/million cells
HEP_rCLint	328.626	Predcited rat hepatic clearance value in µL/million cells
UGT1A1	Yes (77%)	Determines if the compound would be a substrate for the enzyme with 85% accuracy

#### e) Predictive transport profile

Transport proteins play a major role in the body for the delivery of drugs. In silico methods offer a comprehensive dataset of the transportation of drug compounds by using the given structural information. Docetaxel interacts with transport proteins in a variety of ways due to its high lipophilicity and polar group present. Interestingly, 99% of the drug's transportation has been found to be carried out by P-glycoprotein, which is the main transporter responsible for transferring docetaxel across cellular membranes. Furthermore, Table 3 shows that, with differing LogD values, OATP1B1 and the bile salt export pump (BESP) are involved in the transport of docetaxel across cellular membranes and tissues.

**Table 3:** Interaction of DOX with transport protein across cellular membranes.

Parameters	Predicted value	Explanation
P-glycoprotein substrate	Yes (99%)	Determines if te molecular entity may act as a substrate for P-gp and influence the transport or not with 86% accuracy
OATP1B1_Substr	Yes (58%)	The prediction accuracy is 86% overall when determining if the molecule functions as a substrate for OATP1B1 (Yes/No). This prediction evaluates the possibility that the drug molecule will interact with the OATP1B1 transporter and affect the substrate's characteristics, or lack thereof
OATP1B3_Inh	Yes (82%)	With an overall accuracy of 86%, predicts whether the substance has the capacity to block the OATP1B3 transporter (Yes/No). This prediction looks at how well the substance can impede or obstruct the ability of OATP1B3 transporter to function, which affects the inhibitory effects of the molecule
BSEP inhibition	Yes (83%)	Determines if te molecular entity will inhibit the BESP and influence the transport of the drug molecules or not with 90% accuracy
P-glycoprotein inhibition	Yes (88%)	Determines if te molecular entity will inhibit the P-glycoprotein and influence the transport of the drug molecules or not with 88% accuracy

#### f) Predictive toxicity profile

Beyond determining if a drug is safe to use; the main objective of toxicity studies is to find any possible negative effects the drug may have. The main goal of toxicity studies is to determine how pharmacological molecules behave in lab animals and how this may directly affect human health. Moreover, it entails giving large dosages to lab animals in order to identify any hazards that may still exist for humans despite exposure to much lower concentrations^20^. Furthermore, a range of in silico techniques are accessible to forecast the toxicity profiles of previously approved drugs as well as the possible toxicity of new molecules. With the goal of reducing side effects, these tools may help researchers develop effective dose formulations and delivery systems for already-approved drugs.

Predictive data on docetaxel shows that it is not mutagenic, but it is notably toxic to hepatocytes and the reproductive system. The drug induces chromosomal toxicity, which may be related to the many histone proteins that are associated with chromosomes. Docetaxel binds to hepatic microsomes, demonstrating interactions with hepatic proteins that may contribute to the drug's hepatic toxicity. Table 4 shows liver toxicity profile which indicates that docetaxel can raise liver enzyme levels, including ALT and ALP, which are signs of liver damage, without changing levels of AST, GGT, or LDH.

**Table 4:** Predictive toxicity profile of DOX by interaction with various physiological systems.

Parameters	Predicted values	Explanation
Bio-concentration factor	0.025	The partition coefficient between fish tissues and the surrounding aquatic environment is represented by the bio-concentration factor in a steady state, which is also known as the concentration ratio (Cfish/Cwater)
Chromosomal aberration	Non-toxic	With an 80% accuracy rate, predicting whether a drug will cause mutagenic chromosomal abnormalities
hERG filter	No (93%)	Predicts the propensity of the drug molecule to block the hERG potassium channel and cause cardiotoxicity with an 88% accuracy rate
MUT 97 + 1537	Yes	Using the *S. typhimurium* TA97 and/or TA1537 strains, the mutagenicity of the pure drug molecule is predicted. This prediction approach has a general accuracy of 85%
Reproductive toxicity	Toxic (91%)	With a 90% accuracy rate, the model gives a qualitative assessment of developmental and reproductive toxicity
Serum ALT	Elevated	Identifying with 89% accuracy if a molecular entity will raise the levels of the SGPT enzyme
Serum ALP	Elevated	Identifying with 91% accuracy if a molecular entity will raise the levels of the ALP enzyme
Serum AST	Normal	Identifying with 84% accuracy if a molecular entity will raise the levels of the AST enzyme
Serum GGT	Normal	Identifying with 94% accuracy if a molecular entity will raise the levels of the GGT enzyme
Serum LDH	Normal	Identifying with 89% accuracy if a molecular entity will raise the levels of the LDH enzyme

### Liposome preparation and characterization

Phospholipids from camel milk MFGM were effectively obtained via Folch extraction method, ensuing successful development of empty (Lp-CM-ChT80) and docetaxel loaded (Lp-CM-ChT80-DTX) liposomes by deploying the thin film hydration technique. This liposomal synthesis process efficiently yielded Lp-CM-ChT80 and Lp-CM-ChT80-DTX with the molar ratios of 80:10:10 of MFGM to cholesterol to tween 80. Employing passive loading approach, docetaxel was encapsulated into the liposomes successfully.

#### a) Scanning electron microscope (SEM)

**Figure 6:**
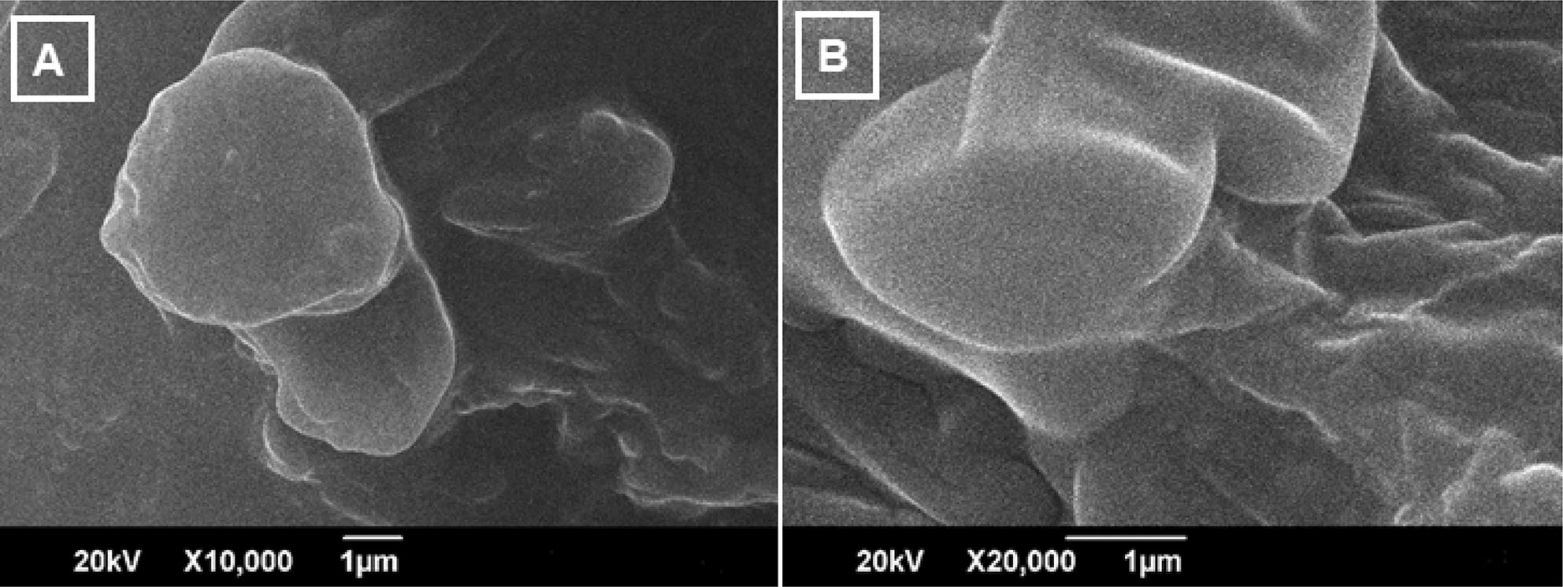
SEM images of Lp-CM-ChT80 (A) and Lp-CM-ChT80-DTX (B) liposome.

According to SEM analysis, Lp-CM-ChT80 were smooth textured and spherical in shape as shown in Fig. 6A. Whereas Lp-CM-ChT80-DTX with relatively bigger sizes showed rectangular morphology as shown in Fig. 6B.

#### b) Fourier transform infrared spectroscopy (FTIR)

**Figure 7:**
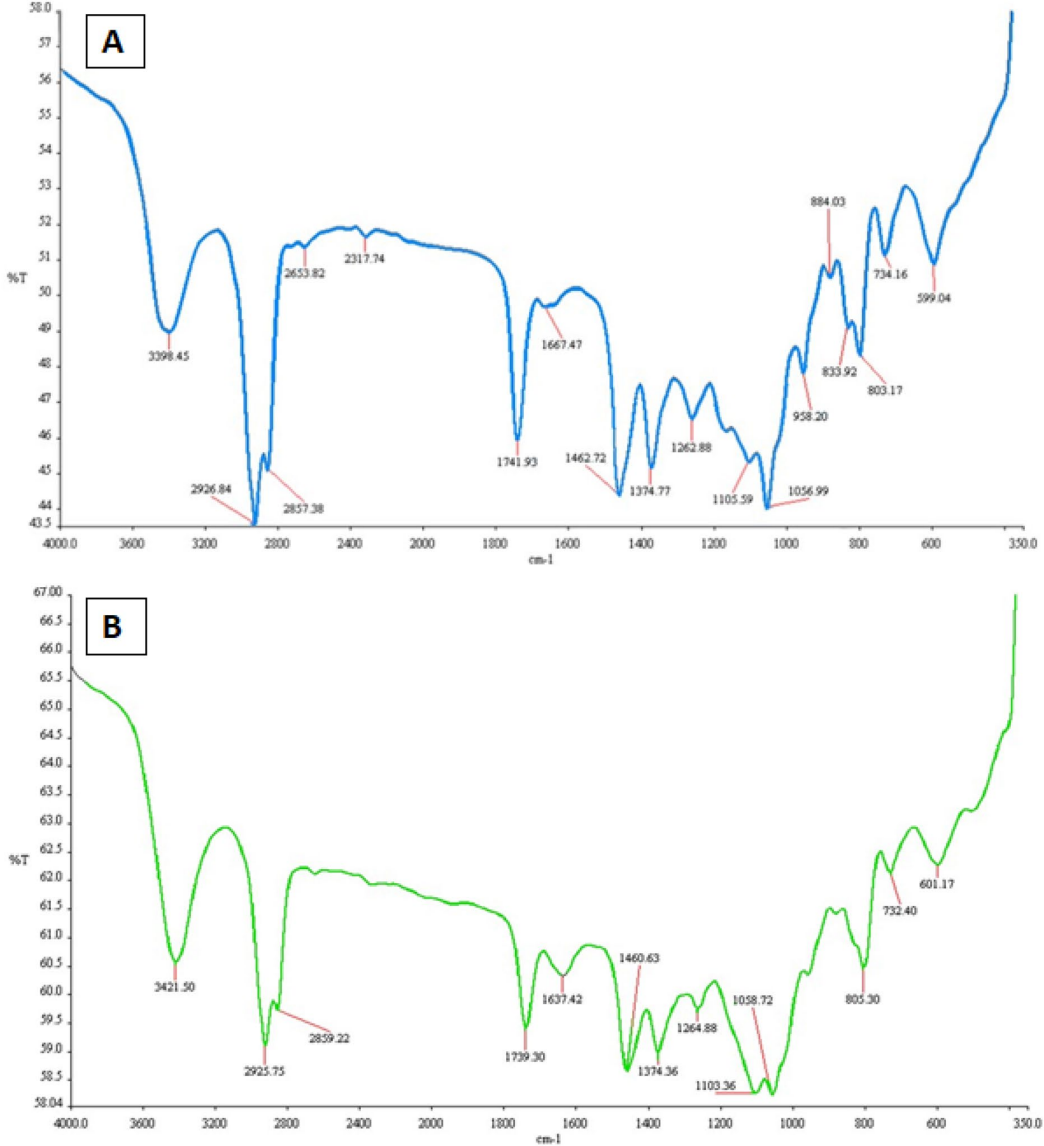
FTIR spectrum of of Lp-CM-ChT80 (A) and Lp-CM-ChT80-DTX (B) liposome.

FTIR was executed for determining the functional groups present in Lp-CM-ChT80 Fig. 7A and Lp-CM-ChT80-DTX liposomes Fig. 7B. The spectrum of Lp-CM-ChT80 demonstrated peaks of MFGM, cholesterol and Tween80 symmetric olefinic C-H stretches at 2857.38 cm^−1^, cholesterol, MFGM and Tween 80 asymmetric aliphatic stretches of C–H at 2926.84 cm^–1^, C=O stretching at 1741.93 cm^−1^, O–H and PO_4_ groups in the range of 500 to 1200 cm^−1^ specifically at 599.04 cm^−1^, 734.16 cm^−1^, 803.17 cm^−1^, 833.92 cm^−1^, 884.03 cm^−1^, 958.02 cm^−1^, 1056.99 cm^−1^ and 1105.59 cm^−1^, whereas the characteristic peaks for O–H bond of cholesterol were conspicuous at 3398.45 cm^−1^.

In the case of Lp-CM-ChT80-DTX liposomes, FTIR spectrum revealed the following peaks of DTX: N–H and O–H stretching at 3421.50 cm^−1^, C=C stretching at 1460.63 cm^−1^, C=O stretching at 1739.30 cm^−1^, C–H stretching at 2925.75 cm^−1^ and 732.40 cm^−1^. Tween80 symmetric CH_2_ peak in this spectrum was present at 2859.22 cm^−1^, while its C=O and O–H stretches were apparent at 1739.30 cm^−1^ and 3421.50 cm^−1^, respectively. The peaks for cholesterol CH_2_ and CH_3_ bonds lied at 2859.2 cm^−1^ and 2925.75 cm^−1^, whereas OH at 3421.50 cm^−1^ as shown in Fig. 7. The spectra for both liposome formulation was same as no major functional group was added or removed during encapsulation process.

#### c) Zeta potential

Malvern Zetasizer Nanos ZS90 (Malvern Instruments, UK) zeta nanosizer was used to measure zeta potential, polydispersity index (PDI) and average particle size of empty and drug loaded liposomes. Readings of diluted liposomes (1:10 ration dilution) were calculated at room temperature. Five different batches were run on zeta nanosizer to obtain average values and for calculation of standard deviation. As shown in Table 5 and Figure S1 (supplementary material), the particle size for empty (Lp-CM-ChT80) was 553.4 nm (A) with PDI of 0.372 which shows very good homogeneity, while zeta potential for empty (Lp-CM-ChT80) was − 22.2 mV (B). For docetaxel loaded (Lp-CM-ChT80-DTX ), the particle size was 836.6 with PDI of 0.088 (C) and zeta potential of − 18.7 mV (D).

**Table 5:** Particle size, zeta potential and PDI of Lp-CM-ChT80 and Lp-CM-ChT80-DTX liposome (*p* < 0.05, n = 5).

MFGM liposomes	Particle size (nm)	Zeta Potential	Polydispersity index
Lp-CM-ChT80	553.4 ± 6	− 22.2 ± 2	0.37 ± 0.2
Lp-CM-ChT80-DTX	836.6 ± 4	− 18.7 ± 0.9	0.088 ± 1

#### d) Drug encapsulation efficiency

**Figure 8:**
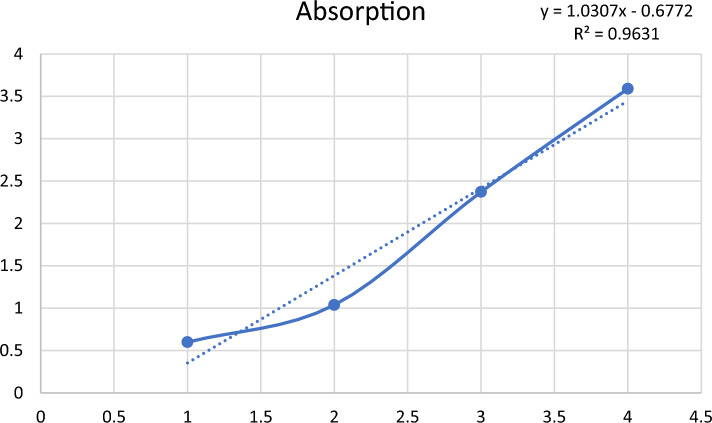
Calculation of entrapment efficiency of DOX in liposomes.

For quantification of DTX loaded in liposomes, UV–Visible spectroscopy revealed 25% entrapment efficiency of DTX in MFGM liposomes as shown in Fig. 8 below.

#### e) In vitro drug release assay

**Figure 9:**
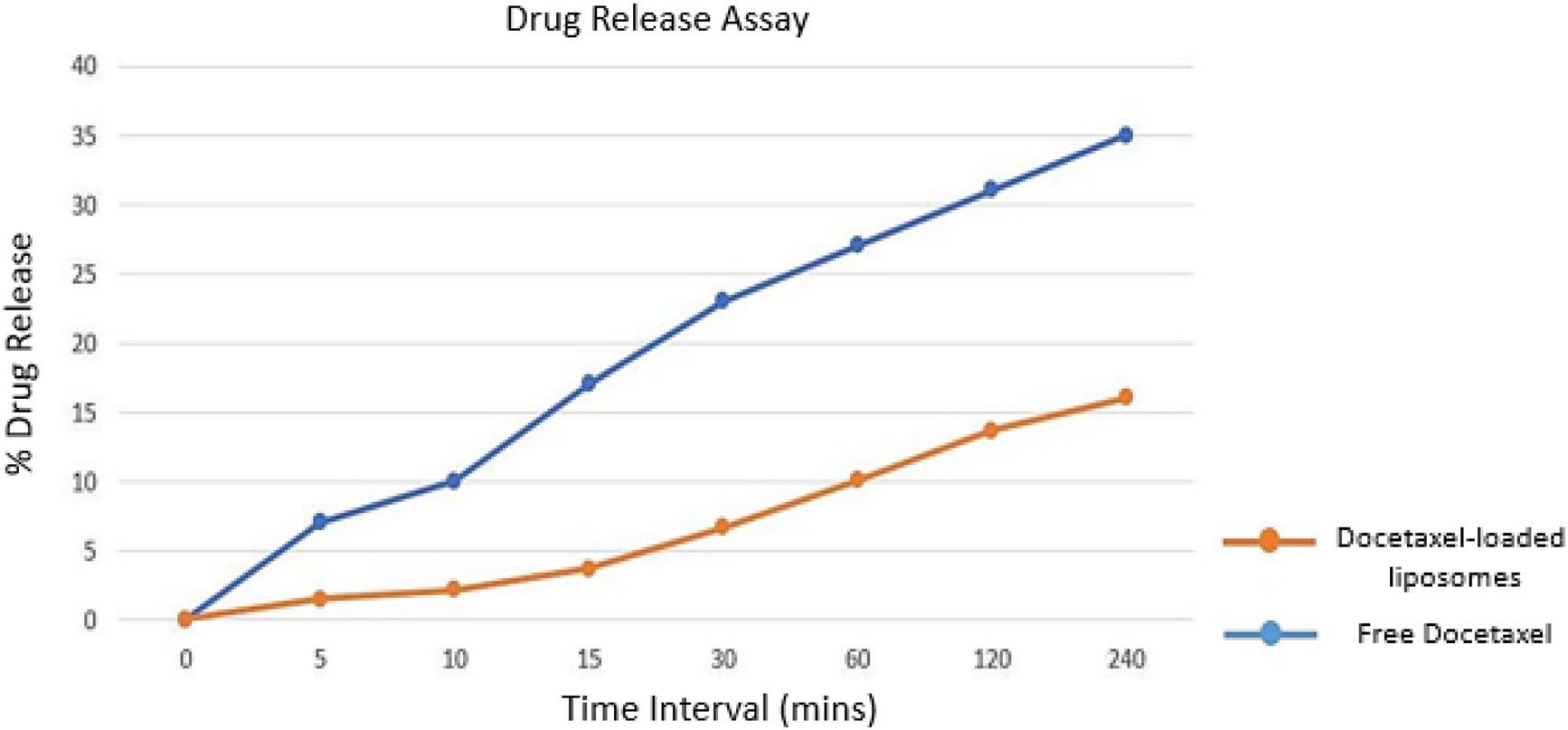
In vitro release profile of Lp-CM-ChT80 and Lp-CM-ChT80-DTX liposome.

In vitro drug release assay was carried out by using dialysis membrane for analyzing the release profile of DOX from Lp-CM-ChT80-DTX liposomes in comparison with free DOX. Figure 9 shows the cumulative release percentage of free DOX and Lp-CM-ChT80-DTX which was measured in PBS at 37 °C.

The measurements were taken over the span of 8 h at 5, 10, 15, 30, 60, 120 and 240-min time intervals. The free docetaxel percent release was 7%, 10%, 17%, 23%, 27%, 31% and 35% at 5, 10, 15, 30, 60, 120 and 240-time intervals, respectively, while in the case of docetaxel-encased liposomes, percent drug release from Lp-CM-ChT80-DTX liposomes was 1.46%, 3.68%, 6.68%, 10.05%, 14.9% and 17% at 5, 10, 15, 30, 60, 120 and 240 time intervals, respectively. The total drug release from Lp-CM-ChT80-DTX liposomes was lower than the drug release from dialysis membrane containing free docetaxel which depicts controlled release.

### In vitro anticancer activity

#### a) Cell morphology analysis

**Figure 10:**
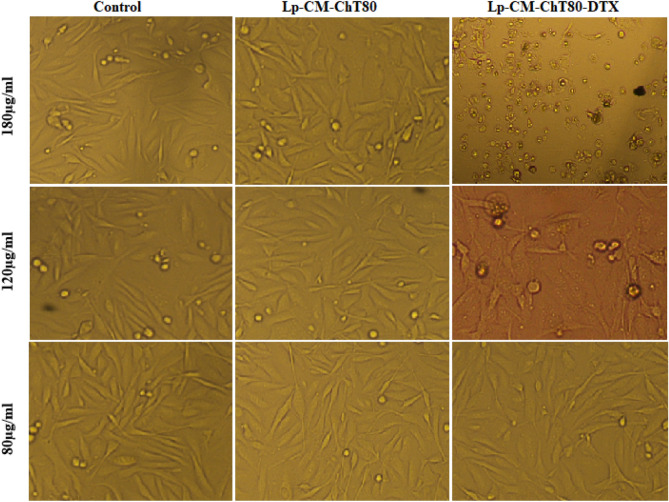
Cell morphology analysis of MDA-MB-231 triple negative breast cancer cell line following treatment with of Lp-CM-ChT80 and Lp-CM-ChT80-DTX liposome.

Changes in cellular morphology indicated the hallmarks of apoptosis upon treatment with with Lp-CM-ChT80-DTX. When MDA-MB-231 cells were treated with 80 µg/ml, 120 µg/ml and 180 µg/ml of Lp-CM-ChT80-DTX for a period of 24 h, cells showed hallmarks of apoptosis such as shrinkage of nuclei, membrane disintegration, and detachment from flask in concentration dependent manner. As shown in Fig. 10, control cells can be compared with Lp-CM-ChT80 and Lp-CM-ChT80-DTX at time intervals 24 h to observe a change in cell morphology and detachment after treatment. The highest toxicity was observed at the time interval of 24 h when treated with 180 µg/ml concentration of Lp-CM-ChT80-DTX, whereas cells treated with same concentration of Lp-CM-ChT80 did not exhibit cytotoxic effect and were comparable to control cells.

#### b) MTT cytotoxicity assay

MTT assay determined the cytotoxic potential of Lp-CM-ChT80 and Lp-CM-ChT80-DTX in TNBC cells MDA-MB-231. The results, as shown in Table 6 revealed that Lp-CM-ChT80 did not have considerable cytotoxicity whereas Lp-CM-ChT80-DTX showed highest cytotoxic effect of 65.2% at concentration of 180 µg/ml while lowest cytotoxicity was 52.3% at concentration of µg/ml. The cytotoxic potential was observed to be dose-dependent. Pure Docetaxel exhibited highest cytotoxicity of 55.1% at 22 µg/ml and lowest cytotoxicity of 30.5% at 5 µg/ml as shown in Table 6.

**Table 6:** MTT cytotoxicity analysis of Lp-CM-ChT80 and Lp-CM-ChT80-DTX liposomes.

S.No	Concentration	Docetaxel	Lp-CM-ChT80	Lp-CM-ChT80-DTX
			% cytotoxicity	
**1**	5 µg/ml	25.5	16.4	52.3
**2**	16 µg/ml	51.5	16.3	60.7
**3**	22 µg/ml	55.1	15.6	65.2

#### c) Trypan blue exclusion assay

Trypan blue exclusion assay was conducted to analyze the cytotoxic effects of Lp-CM-ChT80 and Lp-CM-ChT80-DTX liposome in treated MDA-MB-231 cells. As shown in Table 7 below, no cytotoxic effects were seen in cells treated with Lp-CM-ChT80 liposomes, whereas in cells treated with Lp-CM-ChT80-DTX liposomes, % cell viability increased with increase in concentration. In this treatment group, maximum cell viability of 35 ± 0.12% was observed at a concentration of 10 µg/ml, while the minimum cell viability of 25 ± 0.09% was observed at a concentration of 80 µg/ml.

**Table 7:** Trypan blue exclusion result of % cell viability.

S.No	Concentration	Lp-CM-ChT80-DTX	Lp-CM-ChT80
		% Cell viability	
1	80 µg/ml	25 ± 0.09	80 ± 0.16
2	40 µg/ml	60 ± 0.71	83 ± 0.12
3	10 µg/ml	35 ± 0.12	85 ± 0.51
4	0 µg/ml	89 ± 0.04	89 ± 0.04

### Stability analysis

Initially Lp-CM-ChT80 were transparent and Lp-CM-ChT80-DTX were milky with runny consistency. The viscosity and color were most stable at 4 °C for both the liposomes till 24 h for Lp-CM-ChT80 and 3 h for Lp-CM-ChT80-DTX. At 25 °C, liposomes showed a gradual color and viscosity change over a span of 2 days whereas at 4 °C liposomes showed drastic changes in color and viscosity. After 24 h, liposomes started adhering to the bottom of the petri plate while after 48 h, viscosity increased and the solution turned hazy in appearance. Changes in viscosity were more drastic in Lp-CM-ChT80-DTX as compared to Lp-CM-ChT80. The size of the particles decreased with time. Table 8 and Figure S2 (supplementary material) shows the zeta analysis of Lp-CM-ChT80-DTX after 24 h, it can be seen that the size of Lp-CM-ChT80-DTX changed from 836.6 nm to 680 nm, PDI changed from 0.08 to 0.41, while the zeta potential changed from − 18.7 mV to 4.88 mV. We suggest storing these liposomal nanocarriers in freeze dried form to retain their characteristic size and anticancer potential.

**Table 8:** Particle size, zeta potential and PDI of Lp-CM-ChT80 and Lp-CM-ChT80-DTX liposome (*p* < 0.05, n = 5).

MFGM liposomes	Particle size (nm)	Zeta Potential	Polydispersity index
Lp-CM-ChT80-DTX	680 ± 3	4.88 mV	0.41 ± 1.2

## Discussion

Breast cancer is responsible for high mortality rate among women, among which Triple negative breast cancer is most aggressive form, which least responds to conventional and drugs and is negative for receptors against which most of breast cancer targeting chemotherapeutic agents are designed. Although DTX is an effective chemotherapeutic drug, but its low bioavailability due to lipophilic nature has reduced its therapeutic efficacy in clinical setting. This aspect of DOX was analyzed by using various tools for ADMET prediction of DOX in human body. The results showed that DOX has low permeability, high pre-systematic metabolism, low solubility, and efflux transport which decreases its bioavailability and ultimately therapeutic index. This highlights the need for drug delivery system which can effectively deliver effective chemotherapeutic modalities to TNBC cells^21^. Liposomes as carriers of therapeutic moieties have superb qualities including non-immunogenicity, biodegradability and biocompatibility. Liposomes also offer sustained release of loaded drug with targeted delivery, decreased toxic effects by improving stability of drug and availability in targeted environment^22^. As reported, 1,3-bistertrahydrofuran-2yl-5FU (MFU) encapsulated liposomal formulation was synthesized for targeted uptake and sustained release of drug in pancreatic cancer. In vitro analysis showed enhanced release of MFU and an enhanced anticancer effect on Panc-1 and MiaPaca-2 cancer cell lines, while in vivo studies revealed a superior anticancer effect of liposomes loaded drug as compared to pure drug^23^.

Results similar to our study were reported by another research, in which camel milk liposomes were used for delivery of anticancer drug etoposide (ETP) in fibrosarcoma mouse model. Fibrosarcoma bearing mouse models were treated with different concentrations of etoposide entrapped camel milk phospholipids liposomes (ETP-Cam-liposomes) and etoposide-loaded DPPC (1,2-Dipalmitoyl-sn-glycero-3-phosphatidylcholine) liposomes (ETP-DPPC-liposomes). The results showed that ETP-Cam-liposomes had hampered the progression of tumor growth as compared to ETP-DPPC-liposomes or free ETP^5^. In another study folate targeted and non-targeted DTX conjugates with polyethylene glycol (PEG) were synthesized for enhanced uptake of DTX. For targeting, PEG-DTX conjugates were surface functionalized with folic acid for enhance uptake by folate receptors. In vitro targeting capacity and cytotoxic potential of FA-PEG-DTX were evaluated in CHO and 4T1 breast cancer cell line while in vivo efficacy was studied in was studied in 4T1, breast cancer BALB/c mice models. The particle characterization analysis showed size of 181 ± 10.1 nm with PDI of 0.17. It was concluded that encapsulation increased uptake and retention of DTX by cancer cells while surface functionalization with folic acid further enhanced therapeutic outcomes^24^. Drug release assay showed that at time interval of 120 h, approximately 30% free docetaxel was released in medium while for Lp-CM-ChT80-DTX 4.9% drug was released which proves sustained release of drug from liposomal drug delivery system. This finding is supported by another study which showed that solid lipid nanoparticles loaded docetaxel was released for 24 h (69.4 µg/mL) as compared to free docetaxel which was released at same time (1000 µg/mL)^25^.

This sustained release of drug increases bioavailability of drug increases cytotoxic outcomes and decreases drug dosage required to achieve maximum therapeutic effect. Our results are in accordance with finding reported earlier which stated that lipid enclosed drugs produced enhanced anticancer effect as compared to free drug. The enhanced therapeutic outcomes could also be due to enhanced permeability and absorption of lipid carriers which ultimately increases intracellular accumulation of drug.

## Conclusion

Our study reveals that MFGM derived liposomes are a stable carrier for encapsulation of Docetaxel, enhances its uptake by cellular membrane and gives higher therapeutic outcome. As a future approach these liposomes can be further characterized to gain a better insight into its physiochemical properties, different approaches can be used to make them targeted against specific cancer antigens and to increase its overall entrapment efficiency.

### Supplementary Information

Supplementary Information.

## Data Availability

All data generated or analysed during this study are included in this published article.//////
