# Supplementary material for: In silico ADMET profiling of Docetaxel and development of camel milk derived liposomes nanocarriers for sustained release of Docetaxel in triple negative breast cancer
Source: Sci Rep. 2024 Jan 9;14:912. doi: 10.1038/s41598-023-50878-8 (PMC10776786; doi:10.1038/s41598-023-50878-8)
Supplement: Supplementary file 1 — Supplementary Information. [file 41598_2023_50878_MOESM1_ESM.docx]

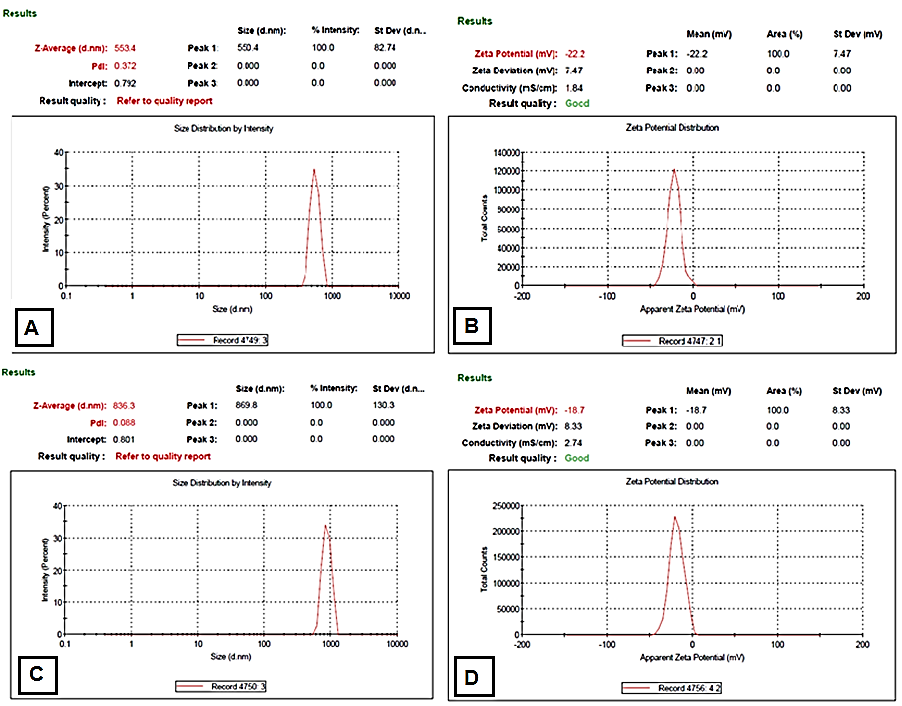
Figure S1: Figure 9: Particle size, PDI (A) and zeta potential (B) of Lp-CM-ChT80 and particle size, PDI (C) and zeta potential of (D) Lp-CM-ChT80-DTX liposome

*
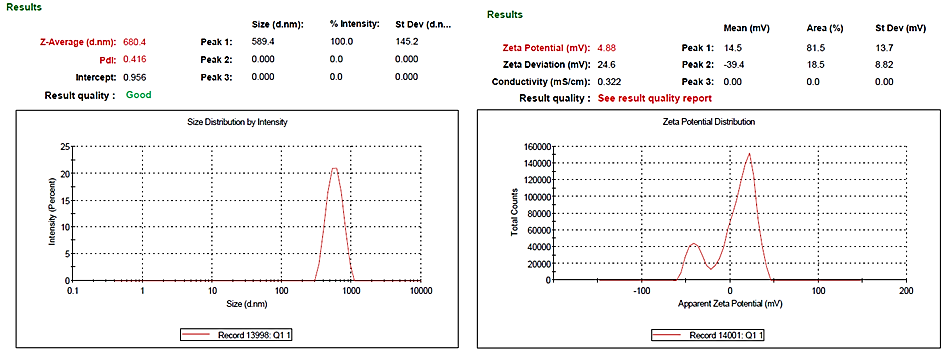
*Figure S2: Stability analysis of Lp-CM-ChT80-DTX after 24 hours following storage at room temperature.
